# Cerebellar Degeneration in Epilepsy: A Systematic Review

**DOI:** 10.3390/ijerph18020473

**Published:** 2021-01-08

**Authors:** Manar Ibdali, Marios Hadjivassiliou, Richard A. Grünewald, Priya D. Shanmugarajah

**Affiliations:** 1 Sheffield Institute for Translational Neuroscience, University of Sheffield, Sheffield S10 2HQ, UK; makibdali1@gmail.com; 2 Academic Department of Neurosciences, Royal Hallamshire Hospital, Sheffield Teaching Hospitals NHS Foundation Trust, Sheffield S10 2JF, UK; m.hadjivassiliou@sheffield.ac.uk (M.H.); r.a.grunewald@sheffield.ac.uk (R.A.G.)

**Keywords:** epilepsy, cerebellar degeneration, ataxia

## Abstract

Introduction: Cerebellar degeneration has been associated in patients with epilepsy, though the exact pathogenic mechanisms are not understood. The aim of this systematic review was to identify the prevalence of cerebellar degeneration in patients with epilepsy and identify any pathogenic mechanisms. Methodology: A systematic computer-based literature search was conducted using the PubMed database. Data extracted included prevalence, clinical, neuroradiological, and neuropathological characteristics of patients with epilepsy and cerebellar degeneration. Results: We identified three consistent predictors of cerebellar degeneration in the context of epilepsy in our review: temporal lobe epilepsy, poor seizure control, and phenytoin as the treatment modality. Whole brain and hippocampal atrophy were also identified in patients with epilepsy. Conclusions: Cerebellar degeneration is prevalent in patients with epilepsy. Further prospective studies are required to confirm if the predictors identified in this review are indeed linked to cerebellar degeneration and to establish the pathogenic mechanisms that result in cerebellar insult.

## 1. Introduction

Epilepsy affects approximately 50 million people worldwide [1] and an estimated 362,000 to 415,000 people in England [2]. Epilepsy is characterised by recurrent and unprovoked seizures [3]. Based on the International League Against Epilepsy (ILAE) Guidelines, epilepsy can be classified by seizure type, epilepsy type, and epilepsy syndrome. Causes of epilepsy include structural, genetic, infectious, metabolic, immune, and unknown factors [4].

Much of what is known about the cerebellum stems from examining the consequences of damage to it [5]. The classical symptoms of cerebellar dysfunction include a broad array of clinical signs, with the most commonly noted being ataxia (a combination of slurred speech, limb incoordination, and gait instability) [6].

The past three decades have witnessed a shift in our understanding of the cerebellum, its function and significance in various neurological conditions [7]. This new understanding, challenges traditional views, suggesting that the cerebellum is associated with the modulation of a variety of cognitive functions including perception, language, memory, and emotion [8,9,10,11].

Cerebellar degeneration refers to the chronic and irreversible loss of neuronal structure and function within the cerebellum [12]; the Purkinje cells are most susceptible. The causes of cerebellar degeneration can be broadly divided into two categories; acquired and genetic [13]. Acquired cerebellar degeneration has been attributed to endogenous or exogenous non-genetic causes [14], such as alcohol abuse and vitamin deficiencies [15], infections of the central nervous system (CNS) [16], autoimmune disorders [17], and primary or metastatic tumours [17], among others. Neuroimaging studies including magnetic resonance (MR) and nuclear medicine techniques such as single-photon emission computed tomography (SPECT) and positron emission tomography (PET) provide structural and functional assessments of cerebellar atrophy [18].

Cerebellar degeneration has been associated with epilepsy [19]. Marcian et al., (2016) [19] postulated a triad of questions; is cerebellar dysfunction a coincidence, consequence, or cause of epilepsy. The authors suggested that cerebellar degeneration can be attributed to cellular damage from seizure activity, the effects of anti-epileptic drugs (AEDs), anoxic-ischaemic injury from seizures, or cerebral hemiatrophy of the cerebellum [20,21,22]. Some patients with epilepsy display variable severity of ataxia as a result of structural cerebellar damage. Sometimes this is merely part of a syndrome where both epilepsy and cerebellar degeneration are features (e.g., mitochondrial diseases), but in many cases, ataxia may be a direct result of seizure mediated cell loss or an adverse effect of certain anti-epileptic medication. Cerebellar damage is also said to result in disinhibition of cerebral epileptic activity. Animal studies have evidenced that cerebellar stimulation induces inhibition of paroxysmal epileptic waves in the cerebrum. Complete ablation of the cerebellum exhibited continuous hypersynchronous activity, in Sprague Dawley rats [23]. Studies linking radiological evidence of cerebellar dysfunction with clinical cerebellar ataxia in patients with epilepsy is also limited. However, the question still remains as to whether atrophy of the cerebellum, causing it to lose its inhibitory effect on cerebral epileptic activity, is the cause or effect of worsening disease progression.

The aim of this study was to systematically review the current literature in order to establish the prevalence of cerebellar degeneration in patients with epilepsy by identifying clinical, neuroimaging and neuropathological characteristics, and its association with epilepsy.

## 2. Methodology

### 2.1. Protocol

This review is registered on the database of dissertation projects for the MSc in Clinical Neurology at the University of Sheffield, UK. This review is not registered on a public database.

### 2.2. Search Strategy

A systematic PubMed search was performed between 27 April 2019 and 11 July 2019. Two Medical Subject Headings (MeSH) were used in the search. Term A was “cerebellar degeneration OR cerebellar atrophy OR cerebellar ataxia OR cerebellar dysfunction”. Term B was “epilepsy OR epileptic OR epilepsia”. No date restrictions were applied in the search strategy.

### 2.3. Inclusion Criteria

In order to be included for the review, studies were required to meet the following criteria:

1. All recruited in the studies were human subjects
2. All patients were diagnosed with epilepsy
3. All studies discussed cerebellar degeneration in relation to epilepsy and/or anti-epileptic drugs
4. All studies investigated clinical and/or neuroimaging and/or pathological findings of cerebellar degeneration in patients with epilepsy
5. The full publication text was written in English

### 2.4. Exclusion Criteria

The following articles were excluded:

1. Animal studies
2. Paediatric studies
3. Case-reports
4. Studies reporting patients with encephalopathy or status epilepticus or post stroke epilepsy
5. Studies detailing cerebellar degeneration in epilepsy caused by another disorder, e.g., alcohol related, autoimmune, mitochondrial
6. Studies where the patient group had been used in multiple studies

**Figure 1:**
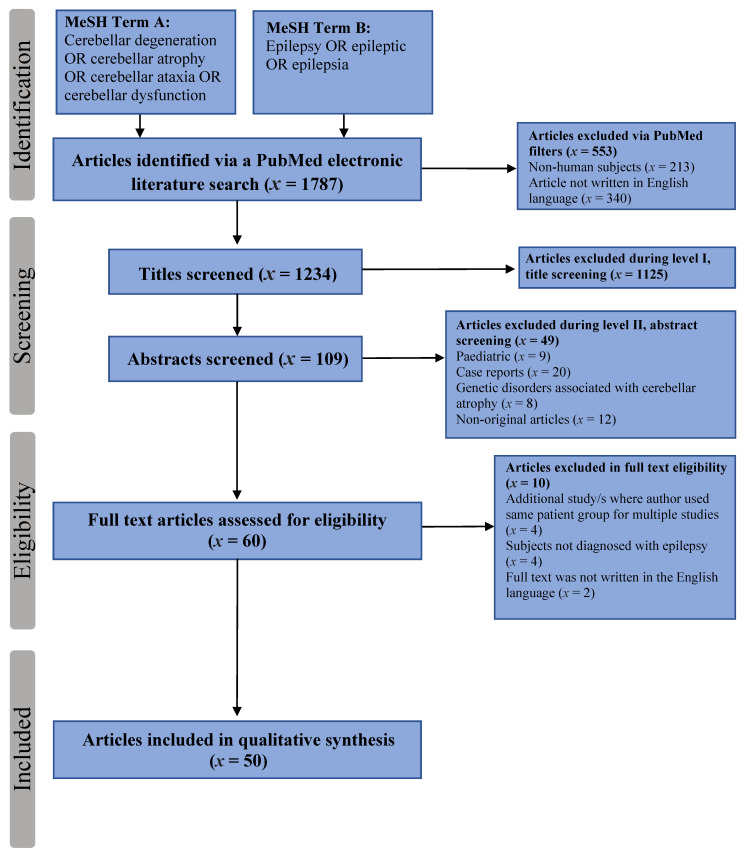
PRISMA chart illustrating the article inclusion and exclusion process. (MeSH: medical subject headings, *x*: number of studies).

All studies were screened and assessed for eligibility by two authors (M.I. and P.D.S.) independently. Details of the inclusion and exclusion process are detailed in Figure 1.

### 2.5. Data Extraction Process

Data was extracted from included studies by a structured coding scheme using Google Sheets in the following format:

1. Study details (author, year of publication, study design, location of study); population demographics (size, age, and gender)
2. Epilepsy characteristics (classification of epilepsy; seizure or epilepsy type, duration of epilepsy, seizure control); anti-epileptic drug (AED) therapy (monotherapy or polytherapy, type of AED at study onset, and duration of treatment).
3. Clinical characteristics of cerebellar degeneration included gait and/or limb ataxia, tremor, nystagmus, and clinical signs of peripheral neuropathy
4. Neuroimaging characteristics included whether cerebellar atrophy was “present” or “absent”; if present whether this was single or bilateral cerebellar hemispheric atrophy, vermian atrophy, or whole cerebellar atrophy); and any focal brain structural changes
5. Neuropathological characteristics on post-mortem included microscopic and macroscopic descriptions of the cerebellum, and when available cause of death.

For the purpose of this review, we referred to the latest 2017 International League Against Epilepsy (ILAE) terminology and definitions of epilepsy [4].

### 2.6. Synthesis of Results

The data extracted was analysed in a chronicle and qualitative manner rather than by quantitative or meta-analysis. This review was performed to comply with the standardised “Preferred Reporting Items for Systematic reviews and Meta-Analyses (PRISMA) guidelines”.

### 2.7. Assessment of Bias

A risk of bias tool was not necessary in this review as none of the included publications were randomised control trials or interventional studies.

### 2.8. Ethical Guidelines

This is based on previous published research and is in accordance with the University of Sheffield’s ethical guidelines.

## 3. Results

### 3.1. Selected Studies

The final PubMed search identified 1787 articles. After limitations were applied (Humans and English Language) 553 articles were excluded before screening. Article screening was performed on two levels: level I, title screening and level II, abstract screening. Level I assessed studies based on title alone. Studies were rejected if they had a definite observable exclusion criterion. In instances where there was no obvious exclusion criterion, abstracts were reviewed. Based on level I screening, 1125 articles were excluded and by level II screening, a further 49 studies were excluded.

A total of 10 articles were excluded in the full text eligibility assessment. Of these, four were studies whereby the patient population had been used in multiple studies of cerebellar degeneration in epilepsy [24,25,26,27]. In such a case, the full text of each study was reviewed and compared against the other. The study judged to have the most data rich and relevant information was subsequently used in the analysis. In relation to the longitudinal studies by Liu et al., [26] and [27], the most recent study was used [28]. Four articles were excluded on the basis that the patient group were not explicitly diagnosed with epilepsy, and two because the full text was written in a language other than English. Finally, a total of 50 studies met the inclusion criteria and constitute our dataset [20,21,22,28,29,30,31,32,33,34,35,36,37,38,39,40,41,42,43,44,45,46,47,48,49,50,51,52,53,54,55,56,57,58,59,60,61,62,63,64,65,66,67,68,69,70,71,72,73,74]. Table 1 and Supplementary Table S2 summarises the study characteristics included.

### 3.2. Epilepsy Characteristics

Thirty-six studies (*n* = 2065) [20,21,28,29,34,35,36,37,39,40,41,44,46,47,48,49,51,52,54,55,56,57,58,59,60,61,62,63,64,65,66,67,68,69,70,74], reported a pooled prevalence of epilepsy classification of either focal (90.3%), generalized (6.5%), or unclassified (3.2%) epilepsy. Patients with TLE represented the vast majority of classifications (*n* = 1503). However, this may be because 21 of these studies exclusively investigated patients with TLE (*n* = 1372) [21,39,47,49,51,54,55,56,57,58,59,60,61,62,63,64,65,66,67,69,70].

### 3.3. Antiepileptic Medication History

For many years phenytoin was considered one of the first-line AEDs for several epilepsy syndromes [75]. Hagemann et al., (2002) [50] speculates that cerebellar damage is a direct consequence of long-term seizures and or AEDs. It can be seen from the data in Table 2 that the AEDs most widely used were phenytoin (44%), phenobarbital (15.5%), carbamazepine (14.3%), and sodium valproate (8.2%). Twenty-three studies commented on whether the pharmacological treatment was monotherapy or polytherapy, with (*n* = 430) and (*n* = 804) patients, respectively [20,29,30,31,32,34,36,37,38,39,41,44,45,46,47,55,61,63,66,69,71,72,73]. There were a lack of studies reporting duration of AED treatment, a factor that could prove essential in determining the contributors to cerebellar degeneration. However, given the well-recognised adverse effects of phenytoin [76], there were only nine studies [20,22,32,33,35,37,41,42,53] that detailed the duration of phenytoin treatment, with a pooled mean duration of 9.3 years, ranging from 0.8 to 67 years. Twenty-nine studies reported on the seizure control of patients with epilepsy [20,21,30,34,35,37,40,42,43,45,46,47,48,49,51,52,54,58,59,60,61,62,65,66,68,69,70,72,74] (*n* = 1303). Of these, eight studies reported 11.2% patients pharmaco-responsive (*n* = 147) and one study detailed 2.1% patients as relapsing-remitting (*n* = 28) to treatment. However, 25 studies observed 86.5% (*n* = 1128) patients as pharmaco-resistant to treatment.

### 3.4. Prevalence of Cerebellar Degeneration

#### 3.4.1. Clinical Characteristics

Fourteen studies reported results from clinical examinations of cerebellar dysfunction, with a total of 788 patients [21,29,30,31,32,33,34,35,36,37,38,39,40,41]. However, quantitative data could only be extrapolated from 13 studies, as some studies merely stated an observation of a clinical cerebellar sign (ataxia, gait ataxia, limb ataxia, tremor, or nystagmus), but did not provide a figure. Additionally, three other studies [31,33,36] reported the presence of ataxia but provided figures for other clinical signs.

The pooled prevalence of the clinical signs of cerebellar dysfunction, from the 13 studies, was 264 (40%) of 657 patients. Nine studies provided information on “ataxia” and provided a pooled prevalence of 156 (24%) patients. Majority of these studies focused on investigating cerebellar atrophy in patients with epilepsy. From these, gait ataxia represented 10%, limb ataxia represented 4%, and the combination of gait and limb ataxia, reported as a clinical sign together, was determined in 14 patients gave a figure of 2%.

Nystagmus was the most frequently observed clinical feature, 10.3% (*n* = 81) in 10 studies that made reference to it [29,31,33,35,36,37,38,39,40,41], whilst tremor represented only 3.4% of the total clinical examination population. However, Specht et al., (1994, 1997) [36,39] contributed the majority of observations on the presence of nystagmus, with 23 and 22 patients, respectively. In both studies, this was related to Carbamazepine intoxication.

Reports on peripheral neuropathy were limited. Two of three studies reported no peripheral neuropathy [33,41] and the other reported peripheral neuropathy in 15 patients [31].

##### Clinical vs. Imaging Characteristics

Of the (*n* = 264) patients documented as having clinical signs of cerebellar dysfunction, 86.7% had cerebellar atrophy via neuroimaging. Volumetric MRI represented (*n* = 67) patients; MR Spectroscopy (*n* = 8); CT (*n* = 28); SPECT (*n* = 4); and PEG (*n* = 122). Moreover, 129 patients displayed vermian atrophy, while 37 also evidenced whole brain atrophy.

##### Clinical vs. Epilepsy and AED Characteristics

Of the total of 788 patients with epilepsy from 13 studies, only nine studies reported the classification of epilepsy, totalling 374 patients [21,29,34,35,36,37,39,40,41]. In this patient group, temporal lobe epilepsy (TLE) represented 38.5%, focal unspecified epilepsy represented 34.5% and focal to bilateral, 2.7%. Generalised epilepsy represented 14.7% and unknown epilepsy 17.6% of patients. These patients were also more likely to use polytherapy as a treatment method, 69.5% (*n* = 453) as opposed to monotherapy, 30.5%, (*n* = 199). The AEDs most frequently used in this group were phenytoin 41.5% (*n* = 555), phenobarbital 16% (*n* = 214), and carbamazepine 10% (*n* = 133), of a total 1337 AEDs administered.

#### 3.4.2. Neuroimaging Characteristics

##### Radiological Techniques and Sequences

Forty-three studies, totalling (*n* = 2451) patients, detailed neuroimaging of the cerebellum [20,21,22,28,29,32,33,34,35,36,37,39,40,41,42,43,44,45,46,47,48,49,50,51,52,53,54,55,56,57,58,59,60,61,62,63,64,65,66,67,68,69,70]. The preferred method of imaging was Volumetric MRI, with 52% studies employing this method. A further 16% used volume-based morphometry (VBM); computed tomography (CT) was used in 10%; SPECT in 6%; diffusion tensor imaging (DTI); and PET represented 4% of studies each; MR Spectroscopy and deformation-based morphometry (DBM) accounted for 2% of studies each. However, due to the variations in result reporting across studies, only 19 reported quantitative data.

##### Imaging Characteristics—Brain Atrophy

Twenty-one studies reported whole brain atrophy, in predominantly white matter regions [22,28,29,32,33,35,36,44,45,47,48,49,50,54,57,60,61,62,65,66,68]. Data from six studies [22,29,32,33,36,47], totalling 349 patients provided a prevalence of 21.2% for whole brain atrophy.

##### Imaging Characteristics—Hippocampal Atrophy

Hippocampal atrophy was identified in fifteen studies (*n* = 1026) [22,28,40,51,54,56,58,59,60,61,62,65,66,69,70]. Of the fifteen studies that reported hippocampal atrophy, all but three [22,28,40], specifically recruited patients with TLE. Quantitative data drawn from eight studies (*n* = 597) [22,28,40,51,58,61,69,70] showed that 77.5% were patients with TLE, and of these 45% (*n* = 269) were observed to have hippocampal atrophy.

In line with previous research [77], we identified widespread grey matter volume (GMV) reductions in the extra-hippocampal regions, including the thalamus and limbic structures, as well as, cerebellar regions [62]. This GMV loss was most pronounced in regions ipsilateral to the epileptogenic focus. That is, reductions in the left cerebellum were observed in left-TLE patients, while right-TLE patients showed bilateral cerebellar reductions [51,56]. However, other studies identified bilateral GMV reductions in the cerebellum of patients with left-TLE and hippocampal sclerosis (HS) [58,65]. Similarly, Oyegbile et al., (2011) [63] found no significant difference of total cerebellar tissue volume, including both GMV and white matter volume (WMV) between left- and right-TLE patients.

##### Brain Atrophy vs. Type of Epilepsy vs. AED

Focal epilepsy comprised 85.5% amongst 1169 patients, while generalised epilepsy represented 10.1% (*n* = 119), and unclassified epilepsy 4.3% (*n* = 51). Amongst the 999 patients with focal epilepsy, 74.6% had TLE. Based on this, it appears there is a moderately greater tendency for whole brain atrophy to be associated with focal epilepsy. The majority of these patients had poor seizure control 85.1% (n = 606). Polytherapy was the treatment modality for 383 patients, compared to 220 using monotherapy. The most prevalent AEDs taken in this group were phenytoin 34% (*n* = 274), carbamazepine 22.5% (*n* = 181), phenobarbital 11.2% (*n* = 90), and barbiturate variants 6.6% (*n* = 53).

##### Imaging Characteristics—Cerebellar Atrophy

The pooled prevalence of cerebellar atrophy based on neuroimaging was 37.5% (*n* = 337), compared to 62.4% (*n* = 561) patients that detected no neuroimaging signs of cerebellar atrophy. The majority of cases report significant cerebellar loss of volume. Some studies suggest a linear relationship between the duration of epilepsy and cerebellar volume loss [21,28,32,49,51,52,54,57,59,63,66]. That is, cerebellar volume decreased as the duration of epilepsy increased, whereas six studies did not find an association [35,37,44,48,50,68].

The loss of cerebellar volume was primarily limited to grey matter. Some studies identified that abnormalities varied across cerebellar hemispheres. Five studies showed bilateral grey matter volume reductions regardless of classification of epilepsy [61,63,65,69,70], Keller et al., (2002 and 2004) [25,51,56] reported that patients with right-TLE focus, presented with bilateral grey matter atrophy, compared with left-TLE patients, who showed ipsilateral reductions in grey matter, using VBM techniques. Using the same modality, Riederer et al., (2008) [60] identified bilateral grey matter reductions in patients with left-TLE. Conversely, Alvim et al., (2016) [69] studied 35 patients whose seizure freedom ranged from 2 to 11 years and observed diffuse grey matter volume reductions in TLE patients, despite prolonged seizure freedom.

Of the forty-three neuroimaging studies identified in our systematic review, five of these used two different neuroimaging techniques each [22,41,48,62,64]. It was difficult to ascertain whether the patients observed one neuroimaging technique or if they were indeed the same patients who underwent both imaging scans and had their results repeated.

Eight studies identified vermian atrophy in their investigations [21,32,35,39,41,42,50,62]. Data from six studies, with a total of (*n* = 353) patients provided a pooled prevalence of 42% (*n* = 148) vermian atrophy. Conversely, Oyegbile et al., (2011) [63] and Marcian et al., (2018) [70] identified an inverse relationship between the cerebellar hemispheres and vermis. That is, the greater the reduction in grey matter volume within the cerebellar hemispheres, the larger the grey matter volume in the anterior lobe of the vermis. This effect was more pronounced in TLE patients who also had hippocampal sclerosis.

#### 3.4.3. Neuropathological Characteristics

Six studies reported cerebellar pathology [22,30,71,72,73,74], totalling 125 patients. Four studies investigated autopsy pathology [22,72,73,74], whilst one exclusively studied brain biopsy [30] and another explored both autopsy and biopsy pathology [74]. Seizure control was only reported in three studies [30,72,74] whereby 62.4% (*n* = 78) patients showed resistance to pharmacological treatment. A total of 72 patients were on polytherapy compared to monotherapy treatment, 39 patients. The most common drug implicated for these pathology patients was phenytoin 30.3% (*n* = 41), followed by phenobarbital 23.7% (*n* = 32) and carbamazepine 16.3% (*n* = 22) (Table 3). Data for epilepsy classification was not available in these studies.

Cerebellar degeneration was reported in five pathology studies [22,30,71,72,74] and detailed a pooled prevalence cerebellar degeneration of 60.4% of (*n* = 101) autopsied and biopsied patients in their investigations. Cerebellar degeneration in this group manifested as significant Purkinje cell loss and a severely atrophied cerebellum. Crooks et al., (2000) [22] found that Purkinje cell loss was higher with increased duration of seizures, although it was difficult to differentiate between seizure duration and age effects.

A high proportion of sudden unexpected death in epilepsy (SUDEP) was reported in these studies. Specifically, 87 (69.6%) deaths were caused by SUDEP in this group [22,72,73,74], while 25 deaths were associated with chronic diseases such as cardiorespiratory insufficiency and coronary diseases [22,73]. One patient committed suicide [74].

The high proportion of SUDEP in these studies can be explained by the fact that Shield et al., (2002) [72] based their neuropathological investigations exclusively on SUDEP brains comparing them to healthy controls. They found a significantly large proportion of SUDEP cases to have neuronal clusters, while the controls displayed both a higher percentage of oligodendroglial clusters and heterotopic neurons in white matter. Both cerebellar and cerebral atrophy was present in these cases. It would be of interest to distinguish possible differences in cerebellar atrophy between SUDEP and patients with epilepsy that died of other causes.

## 4. Discussion

To our knowledge, this is the first systematic review investigating cerebellar degeneration in patients with epilepsy. We aimed to identify patterns of cerebellar degeneration in relation to clinical, neuroimaging and pathological characteristics. We also set out to identify any links between cerebellar degeneration with seizure type and AEDs.

Through qualitative synthesis of our dataset, we identified several factors that are associated with cerebellar degeneration across clinical, neuroimaging and pathology studies. Supplementary Table S1 provides a summary of the key findings. The three main factors are (1) patients who have temporal lobe epilepsy, (2) patients with poor seizure control, and (3) patients treated with phenytoin.

### 4.1. TLE and Cerebellar Degeneration

Although cerebellar degeneration was evident in patients with epilepsy regardless of epilepsy classification, it was most prevalent in patients with TLE. Temporal lobe epilepsy is the most common pharmaco-resistant epilepsy in adults [78] and frequently requires resective surgery of the affected temporal lobe [79]. It is characterised by seizures that arise anywhere within the temporal lobe. In TLE, the seizure originates in the limbic structures within the temporal lobe, more specifically, the hippocampus [77,80]. Hippocampal sclerosis is a hallmark feature of drug-resistant TLE and characterised by neuronal death, gliosis, and atrophy [81].

Additionally, Bonilha et al., (2010) [62] using DTI and VBM techniques, identified a relationship between hippocampal deafferentation and regional brain atrophy in TLE patients; this hippocampal fibre disconnection may be partially responsible for grey matter atrophy in temporal, basal nuclei, and cerebellar areas. The presence of HS, however, does not necessarily imply cerebellar atrophy. In other words, cerebellar volume loss was identified, even in the absence of HS [64,65] and prolonged seizure freedom did not prevent cerebellar atrophy [69]. These findings are supportive of the growing evidence that the distribution of atrophy within the brain of TLE patients, preferentially affects brain structures that are anatomically and functionally connected to the hippocampus and cerebellum [62]. However, the high prevalence of pharmaco-resistant TLE patients with cerebellar degeneration may reflect the lack of routine neuroimaging in drug-responsive patients, patients with generalised epilepsy, and those with no clinical deficits [82].

### 4.2. Pharmaco-Resistance and Cerebellar Degeneration

Epilepsy treatment has evolved significantly, from the traditional use of bromides, to the modern era in which we now utilise various treatment modalities. These include medications, implantable devices, non-pharmacological therapies, and surgery [83]. AEDs remain the mainstay treatment options for epilepsy, with approximately 50% of patients becoming seizure free with monotherapy [84]. However, only 11% of patients who fail to achieve seizure freedom with monotherapy, due to inefficacy, experience a reduction with a second treatment option [85]. These findings suggest that the probability of treatment response reduces with each treatment failure [86,87]. Although, the terms “refractory”, “intractable”, and “pharmaco-resistant” epilepsy are used interchangeably. The ILAE defines “drug-resistant epilepsy” as a failure to achieve and maintain seizure freedom despite trialling two AEDs that are well tolerated, in terms of side effects, whether as a monotherapy or polytherapy [88].

Approximately 30 to 40% of patients with epilepsy fail to achieve adequate control of seizures, despite the use of multiple AEDs and different treatment therapies [89]. Our review identified an exceedingly high prevalence of drug-resistant patients that demonstrated cerebellar degeneration (87.2%). This is consistent with the general consensus that drug-resistant epilepsy has more profound brain damage than drug-responsive epilepsy [77,90]. Factors that induce cerebellar degeneration in pharmaco-resistant patients remain to be determined, whether this is related to seizure mediated cell loss or from direct effects of status epilepticus or from the use of multiple AEDs at high serum concentrations.

Patients responsive to AED treatment were also observed to have cerebellar degeneration, indicating that multiple factors may contribute to the development of cerebellar degeneration in epilepsy [61]. However Bilevicius et al., (2010) [61] found that cerebellar degeneration in patients with drug-resistant and relapsing-remitting seizures was more profound and widespread than drug-responsive epilepsy. Despite this, there is a paucity of studies evaluating the influence of seizure control on cerebellar degeneration. There may be bias in performing neuroimaging only in patients with pharmaco-resistant epilepsy. Studies evaluating cerebellar degeneration should include both patients with uncontrolled epilepsy as well as stable epilepsy.

### 4.3. Phenytoin and Cerebellar Degeneration

The correct choice of an AED is crucial to therapeutic success. Our review identified an increased number of patients using phenytoin. Since its introduction to epilepsy treatment in 1938, phenytoin had become the most extensively prescribed and studied anticonvulsant [91]. It acts by blocking voltage-dependent sodium channels, suppressing the propagation of discharges from seizures. However, due to the narrow therapeutic index of phenytoin; its risks of acute over-dosage and the toxicity effects of its chronic use [92], phenytoin is now gradually becoming less popular. Long-term use of phenytoin has already been associated with significant risk of cerebellar degeneration and ataxia, which can persist long after discontinuation [7].

The chronic effects of phenytoin are often difficult to discriminate from that of the seizures [43]; as the analyses are complicated by the fact that patients with severe epilepsy are also on higher doses of medication [50]. Despite this, our findings lend confidence to the notion that chronic phenytoin use, whether in the therapeutic range or not, is an important factor of cerebellar degeneration. As we are now in an era whereby there is a variety of viable AED options, it would be interesting to compare phenytoin-exposed patients with those that have not been exposed [20]. Phenytoin use in epilepsy may be much higher than any other AED; thus, the connection with cerebellar degeneration may simply be because it is used frequently. Proportionally more patients on phenytoin develop ataxia than patients on other AED. Further studies should focus in comparing phenytoin as monotherapy and/or combination therapy with other AED and treatment duration in determining its link with cerebellar degeneration.

### 4.4. Clinical Characteristics of Cerebellar Degeneration

Previous research has demonstrated a weak relationship between the clinical manifestations of cerebellar dysfunction and neuroimaging characteristics of cerebellar atrophy [93]. In our review, cerebellar degeneration by assessment of cerebellar atrophy was evidenced in 86.7% of patients with epilepsy. Whether this was related to the toxic effects of AEDs or drug-resistant epilepsy or the use of polytherapy as a treatment mechanism; or the combination of all three, remains unclear. Although, studies evaluated on their own merits did not find a link between cerebellar atrophy in neuroimaging and the clinical evidence of ataxia, Shanmugarajah et al., (2018) [41] identified significantly smaller cerebellar volumes in patients with phenytoin related ataxia, compared with patients taking phenytoin without ataxia. These findings suggest a possible threshold by which cerebellar atrophy may manifest clinically with cerebellar signs.

Although studies have postulated that ataxia is a direct result of cerebellar atrophy [55,57], others have found no association between the presence or absence of cerebellar atrophy on the clinical manifestation of ataxia [33,37], or of the severity of symptoms [35].

Ataxia, in all available studies, was associated with the cerebellar atrophy. Reports on peripheral neuropathy were limited, therefore no conclusions could be derived in this regard. Although we aimed to examine neuropathological studies in our review, these were limited in our search.

## 5. Limitations

Several confounding variables can be drawn from the population demographics. Some studies included patients with additional features such as headache and migraine [20,32,42,49], history of alcoholism [52,72], and intellectual disabilities [29,36,66]. Some studies were retrospective. Studies that employ this method are subject to various risks of validity; because they rely on the accuracy of written medical records, controlling for bias and confounding variables is challenging, and establishing cause and effect proves difficult [94]. In addition, the retrospective feature of these studies may introduce selection bias [95], as some studies recruited from specialised centres or hospital clinics, with a predilection towards chronic, severe, and often drug-resistant epilepsy [50].

Some studies used a cross-sectional design, which cannot infer causality. This is especially important where the age of onset and duration of epilepsy are concerned [63]. As such, inferences on disease progression over time, in particular, the effects of chronic epilepsy on changes in brain morphology, are limited [28]. This is because subtle changes in brain structure are difficult to detect over the large inter-patient variability [27]. We aimed to determine the clinical characteristics of cerebellar dysfunction in patients with epilepsy. However, the presence of it does not necessarily indicate cerebellar degeneration as similar signs can be seen in AED intoxication. Future studies defining clinical characteristics should exclude intoxication as a cause by performing serum AED levels simultaneously.

There were significant variations across neuroimaging studies [57]. Firstly, due to a lack of well-defined criteria, diagnosis of atrophy is often based on manual tracing of the cerebellum and subjective interpretations [50]. Specht et al., (1997) [21] identified that MRI ratings of cerebellar atrophy differed by 37% between two independent neurologists, thus introducing inter-rater bias [37]. Secondly, emerging literature suggests that volumes obtained from volumetric MRIs vary according to manufacturer [82], scanner type, field strength, and slice thickness [96]. Taking these points into consideration, future studies should consider using 1.5 Tesla magnet and thinner slices to obtain an accurate and reliable measure of the cerebellum [96].

Following the work of the ILAE, the definitions and criteria for epilepsy diagnosis are evolving [97]. It is thus not possible to determine whether we have reported accurate prevalence rates for different epilepsies. Due to the broad publication dates of included studies, spanning just over a four-decade period and variation, we cannot be certain how stringently the ILAE guidelines were followed or if they were followed at all. The primary concern of defining epilepsy is not straight forward. Where definitions were provided, these consisted of standard definitions established by the ILAE criteria, which would have been relevant for that time period. Whereas, other definitions specified variable diagnostic criteria, in relation to clinical evaluation, EEG recordings and neurological examinations, such as number of seizures and treatment with AED medications.

The prevalence of phenytoin use overall compared to other AEDs may have influenced the results. Phenytoin was consistently the highest used AED in all sections of our review.

The articles used in this review were retrieved via a search in a single electronic database, PubMed. Bramer et al., (2017) [98] recommended systematic reviews should utilise a combination of MEDLINE, Embase, Web of Science Core Collection and Google Scholar databases, in order to achieve >95% literature recall.

## 6. Future Studies

As well as considering the limitations in our review, future studies would benefit from a more stringent exclusion criteria, excluding patients with alcohol misuse; those cognitively impaired and patients with epilepsy with structural lesions. Furthermore, research should focus on conducting multicohort longitudinal studies. The study should be of a sufficient sample size and with adequate power, in order to identify variations of clinical significance, and unequivocally differentiate disease progression from natural age-related atrophy. Longitudinal studies will enable detection of physical changes of cerebellar degeneration between patients with epilepsy and healthy controls [99]. Studies should also appreciate the heterogeneous nature of epilepsy, the various different AEDs including newer agents, and consider investigating cerebellar degeneration in relation to patients with seizure freedom to identify the extent of brain damage from seizures themselves.

## 7. Conclusions

In summary, cerebellar degeneration is prevalent in patients with epilepsy. This review identified a trio of predictors of cerebellar degeneration. Patients with temporal lobe epilepsy, patients with drug-resistant epilepsy and those treated with chronic use of phenytoin were most susceptible to cerebellar degeneration. Whether cerebellar atrophy is a predisposing factor for cerebellar ataxia in patients with epilepsy is inconclusive, due to the lack of clinical symptom reporting. Further prospective studies are required to confirm if the predictors identified in this review are indeed linked to cerebellar degeneration and to establish the pathogenic mechanisms that result in cerebellar insult.

## Figures and Tables

**Table 1:** Summary of study characteristics included.

Total Number of Studies Included in this Review	50
Types of publications (%)	
Cross-sectional	18 (36)
Correlational	14 (28)
Case-Control	10 (20)
Cohort	7 (14)
Longitudinal	1 (2)
Year of publication	
Range	1976–2018
Number of publications per decade	
1970–1980	5
1981–1990	3
1991–2000	17
2001–2010	14
2011–present	11
Number of patients with epilepsy studied	
Total number of patients with epilepsy	2826
Mean number of patients per study (SD)	56.5 (54.6)
Range	3–248
Median	41

SD = standard deviation.

**Table 2:** Number of patients on monotherapy and polytherapy anti-epileptic drug (AED) medications at study onset.

Type of Treatment (%)		
Monotherapy	430 (34.8)	
Polytherapy	804 (65.2)	
AED Type	Number of Patients	(%)
Phenytoin	914	(44)
Phenobarbital *	324	(15.5)
Carbamazepine	299	(14.3)
Sodium Valproate/Valproic Acid	167	(8.25)
Primidone	119	(5.88)
Diazepam	74	(3.66)
Acetazolamide	44	(2.17)
Lamotrigine	30	(1.48)
Nitrazepam	25	(1.24)
Gabapentin	22	(1.09)
Clonazepam	19	(0.94)
Vigabatrin	15	(0.74)
Sulthiamine	14	(0.69)
Topiramate	11	(0.54)
Trimethadione	5	(0.25)
Ethosuximide	3	(0.15)
Fluoxetine	2	(0.10)
Zonisamide	2	(0.10)
Felbamate	2	(0.10)
Haloperidol	1	(0.05)
Tiagabine	1	(0.05)

* Includes barbiturate variants.

**Table 3:** Table demonstrating the number of patients taking each anti-epileptic drug (AED) for seizure control based on neuropathological studies.

AED Type	Number of Patients	(%)
Phenytoin	41	(30.37)
Phenobarbital	32	(23.7)
Carbamazepine	22	(16.3)
Clonazepam	9	(6.67)
Sodium Valproate/Valproic acid	7	(5.19)
Primidone	7	(5.19)
Lamotrigine	6	(4.44)
Vigabatrin	5	(3.7)
Diazepam	4	(2.96)
Ethosuximide	1	(0.74)
Acetazolamide	1	(0.74)

(Note: retrospective data from medical reports).

## Data Availability

Data presented in this study are based on previous published research referenced in this article.

## Supplementary Materials

The following are available online at https://www.mdpi.com/1660-4601/18/2/473/s1, Table S1: Summary of main results; Table S2: Summary of study types included.
