# Supplementary material for: Cerebellar Degeneration in Epilepsy: A Systematic Review
Source: Int J Environ Res Public Health. 2021 Jan 8;18(2):473. doi: 10.3390/ijerph18020473 (PMC7827978; doi:10.3390/ijerph18020473)
Supplement: Supplementary file 1 [file ijerph-18-00473-s001.pdf]

Supplementary Material

**Total number of articles included in this review**	50
**Total number of patients with epilepsy**	2826
**Total number of patients with temporal lobe epilepsy (TLE)**	1503
**Total number of pharmacoresistant patients**	1128
**Total number of patients using phenytoin**	914

Table S1. Summary of main results

**Study design**	**Cross-sectional**	**Correlational**	**Cohort**	**Case Control**	**Longitudinal**
**Author, year of publication**	Shanmugarajah et al, 2018	Bohnen et al, 1998	McDonald et al, 2008	Hellwig et al, 2013 ‡	Liu et al, 2005
	Alhusaini et al, 2012	Botez et al, 1988	Specht et al, 1997b	Bekkelund et al, 1996 ‡	
	Bileviciuis et al, 2010	Ballenger et al, 1982	Iivanainen et al, 1977 ‡	Ney et al, 1994 ‡	
	Bonilha et al, 2004	Crooks et al, 2000	Savic et al, 1996	Sandok et al, 2000 ‡	
	Hagemann et al, 2002	De Marco et al, 2003	Rambeck et al, 1992	Jibiki et al, 1993 ‡	
	Bonilha et al, 2010	Benvenuti et al, 1992	Recio et al, 2007 ‡	Rajjoub et al, 1976 ‡	
	Hermann et al, 2004	Luef et al, 1996	Nakazawa and Ohkawa, 1980	Blum et al, 1998 ‡	
	Keller et al, 2004	Park et al, 2015		Robertson et al, 1979	
	Lee et al, 2003	Salcman et al, 1978		Sequeira et al, 2013 ‡	
	Misra et al, 1997	Scanlon et al, 2011		Shields et al, 2002 ‡	
	Oyegbile et al, 2011	Specht et al, 1997a			
	Marcian et al, 2018	Szabo et al, 2006			
	Riederer et al, 2008	Luef et al, 1994			
	Theodore et al, 1987	Specht et al, 1994			
	Hermann et al, 2005				
	Alvim et al, 2016				
	Keller et al, 2002				
	Savic and Thorell, 1996				

‡ Retrospective studies

Table S2. Summary of study types included
